# Serotonin is the main tryptophan metabolite associated with psychiatric comorbidity in abstinent cocaine-addicted patients

**DOI:** 10.1038/s41598-019-53312-0

**Published:** 2019-11-14

**Authors:** Pedro Araos, Rebeca Vidal, Esther O’Shea, María Pedraz, Nuria García-Marchena, Antonia Serrano, Juan Suárez, Estela Castilla-Ortega, Juan Jesús Ruiz, Rafael Campos-Cloute, Luis J. Santín, Fernando Rodríguez de Fonseca, Francisco Javier Pavón, María Isabel Colado

**Affiliations:** 1 Unidad Gestión Clínica de Salud Mental, Instituto de Investigación Biomédica de Málaga (IBIMA), Hospital Regional Universitario de Málaga, Universidad de Málaga, Málaga, Spain; 2 Departamento de Psicobiología y Metodología de las Ciencias del Comportamiento. Facultad de Psicología, Universidad de Málaga (UMA), Málaga, Spain; 3 Departamento Farmacología y Toxicología, Facultad de Medicina, Universidad Complutense, Madrid, Spain; 4 Centro Provincial de Drogodependencias, Málaga, Spain

**Keywords:** Biomarkers, Medical research

## Abstract

The lack of effective treatments and a high rate of relapse in cocaine addiction constitute a major health problem. The present study was conducted to examine the expression of tryptophan-derived metabolites in the context of cocaine addiction and psychiatric comorbidity, which is common in addicted subjects. Abstinent patients with cocaine use disorder (CUD) and control subjects were recruited for a cross-sectional study. Participants were assessed with a semi-structured diagnostic interview (PRISM) based on DSM-IV-TR for substance and mental disorders. Plasma concentrations of tryptophan metabolites and their association with relevant CUD-related variables and psychiatric comorbidity were explored. We observed decreased plasma kynurenic acid concentrations in the cocaine group, however no associations between CUD-related variables and tryptophan-derived metabolites were found. In contrast, 5-HT concentrations were increased in CUD-patients and the diagnosis of different psychiatric disorders in the cocaine group was related to higher plasma 5-HT concentrations compared with non-comorbid patients. Therefore, while changes in plasma kynurenic acid concentrations appear to be directly associated with lifetime CUD, changes in 5-HT concentrations are associated with psychiatric comorbidity. These results emphasize the need to find potential biomarkers for a better stratification of cocaine-addicted patients in order to develop therapeutic approaches to prevent cocaine relapse.

## Introduction

Cocaine dependence is a substantial contributor to global disease burden and is associated with high social costs^1^. Consequently, chronic cocaine use is a major health problem affecting millions of people, a situation further complicated in subjects with additional psychiatric conditions^2^. Research has been carried out for years trying to unravel the mechanism of action underlying the addictive potential of cocaine. However, despite the progress made in this sense, to date there are no effective treatments available^3^.

Cocaine inhibits dopamine, noradrenaline and serotonin (5-HT) reuptake and while the dopaminergic system has been most-directly linked to the reinforcing aspects of the drug, the role of 5-HT remains unclear. Animal studies implicate 5-HT receptors in cocaine-seeking and -taking behavior^4^ and genetic ablation of the 5-HT transporter (SERT) in rodents is associated with alterations in the rewarding, psychomotor stimulant and motivational properties of cocaine^5–7^. On the other hand, cocaine withdrawal is associated with psychiatric syndromes such as depression^8,9^, which are, in turn, associated with reduced brain 5-HT concentrations although the role of the neurotransmitter in comorbidity with mental disorders is still the object of study.

Tryptophan (TRP) is an essential amino acid that is also the precursor of multiple bioactive products. A small proportion of TRP is used for 5-HT synthesis while most is metabolized through the kynurenine (KYN) pathway producing KYN and biologically-active metabolites, including kynurenic acid (KA), 3-hydroxykynurenine (3HK) and quinolinic acid (QA). While 3HK and QA are neurotoxic metabolites, KA is thought to be involved in neuroprotection. Because 5-HT is a relevant neurotransmitter in the pathogenesis of mood disorders, the balance between neurotoxic and neuroprotective metabolites of TRP has also been proposed to be involved in the genesis and course of these mood disorders^10^. Recently, pharmacological increases of KA have been proposed as a novel strategy for treating human marijuana and nicotine dependence^11,12^, and it has been suggested that KA may play a role in relapse-like situations since its increment abolished cocaine-seeking behavior^13^.

Two rate-limiting enzymes, tryptophan 2,3-dioxygenase (TDO) and indoleamine 2,3-dioxygenase (IDO), control TRP degradation through the KYN pathway. Interestingly, IDO is tightly associated with the modulation of the immune system. Previous results from our laboratory have established that subjects with cocaine use disorder (CUD) (i.e., cocaine abuse and/or dependence) have altered circulating concentrations of inflammatory cytokines and chemokines, reflecting a disrupted immune system response to cocaine^14^. Thus, alterations of the KYN pathway might contribute to cocaine-induced changes in inflammatory responses that are linked to cocaine symptom severity.

In this study, we explored the hypothesis that cocaine-induced alterations in TRP-derived metabolites in the plasma of cocaine-addicted subjects are persistent during abstinence and might represent relevant clinical indicators of variables related to cocaine addiction and psychiatric disorders, which might help in the stratification of CUD-patients in order to improve the efficacy of treatments. The aim of this study was to investigate the effect of a history of cocaine use on the expression of the two main pathways of TRP metabolism, the 5-HT and the KYN pathways in plasma. To this end, plasma concentrations of TRP, KYN, 5-HT, KA and QA were quantified in abstinent cocaine-addicted patients and control individuals. Furthermore, given the close relationship between cocaine addiction and the presence of mental illness, we explored the possible association between changes in TRP metabolites and psychiatric comorbidity.

## Results

### Participants and socio-demographic variables

A total of 160 participants were confirmed for this study and grouped into cocaine (N = 100) and control (N = 60) groups. As shown in Table S1, CUD-participants were mainly males (p < 0.001) and the average cocaine-addicted participant was a 35 year-old male with a BMI of 25 (weighing 76–79 kg). The cocaine group had significantly lower educational level and higher rate of unemployment than controls (p < 0.001) because the control group was predominantly recruited from sanitary and academic staff.

### Plasma concentrations of TRP-derived metabolites

The effect of lifetime CUD on the plasma concentrations of metabolites related to TRP metabolism (concentrations and ratios) in the sample was investigated using ANCOVA with ‘diagnosis of CUD’ as main factor and controlling for ‘sex’, ‘age’ and ‘BMI’. A base-10 logarithmic transformation of estimated marginal means was conducted for KA, 5-HT and ratios.

**Figure 1:**
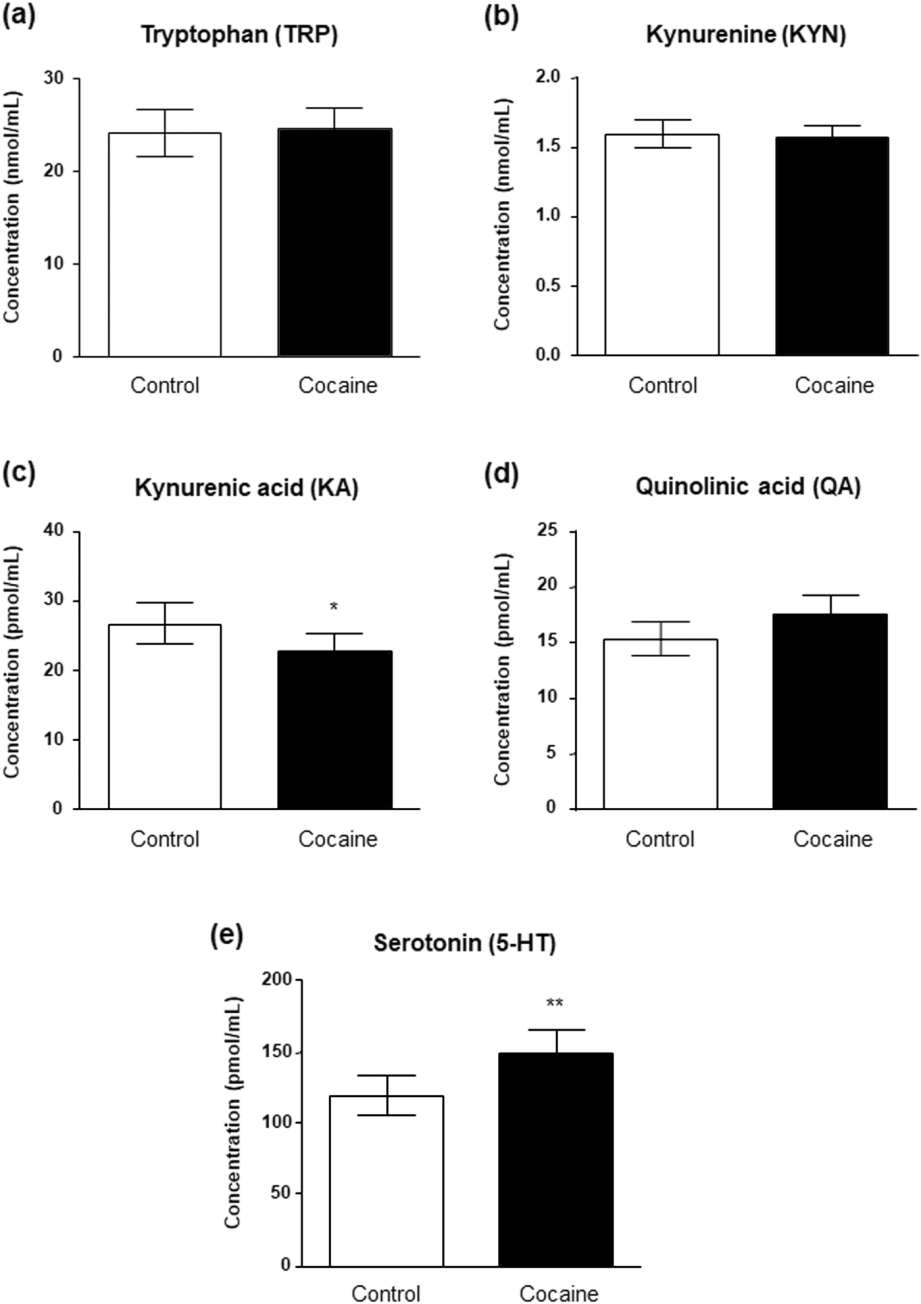
Plasma concentrations of TRP-derived metabolites in abstinent CUD-patients and control subjects. Plasma concentrations of **(a)** TRP, **(b**) KYN, **(c**) KA, **(d)** QA and **(d)** 5-HT were determined in the cocaine group and the control group. Bars are estimated marginal means and 95% CI. Data were analyzed by ANCOVA. (*)p < 0.05 and (**)p < 0.01 denote significant differences compared with the control group.

As shown in Fig. 1, concentrations of TRP, KYN, KA, QA and 5-HT were differentially affected. Thus, although plasma concentrations of TRP (Fig. 1a), KYN (Fig. 1b) and QA (Fig. 1d) were not affected by ‘diagnosis of CUD’, we observed a main effect of this factor on KA (F_(1,154)_ = 4.045, p = 0.045; Fig. 1c) and 5-HT (F_(1,153)_ = 7.996, p = 0.005; Fig. 1e) concentrations. Indeed, KA concentrations were significantly lower in the cocaine group than in the control group [22.86 (95% CI = 20.70–25.23) pmol·mL^−1^ and 26.61 (95% CI = 23.82–29.72) pmol·mL^−1^, respectively] whereas 5-HT concentrations were significantly increased [148.81 (95% CI = 133.86–160.10) pmol·mL^−1^ and 118.76 (95% CI = 105.59–133.55) pmol·mL^−1^, respectively].

Among confounding variables, the ‘BMI’ was strongly related to KYN concentrations (F_(1,154)_ = 7.013, p = 0.009). In fact, correlation analysis revealed a positive association between BMI and KYN concentrations (r =  + 0.318, p < 0.001) in the total sample (Fig. S1). Additional correlation analyses between BMI and KYN concentrations were conducted according to ‘diagnosis of CUD’ and a significant correlation was observed only in the cocaine group (r = + 0.378, p < 0.001).

Table S2 shows KYN/TRP, KA/KYN, QA/KYN and 5-HT/TRP ratios. Whereas KYN/TRP and QA/KYN ratios were not affected by ‘diagnosis of CUD’, KA/KYN and 5-HT/TRP ratios were significantly affected (F_(1,154)_ = 5.238, p = 0.022; and F_(1,152)_ = 3.986, p = 0.046); respectively). Thus, KA/KYN ratio was lower in the cocaine group. In contrast, 5-HT/TRP ratio was higher in the cocaine group compared with controls.

### Characteristics related to pattern of cocaine use and lack of effects on TRP metabolism in abstinent CUD-patients

The cocaine group was characterized using variables related to pattern of use as shown in Table 1. Cocaine users were recruited in abstinence from cocaine for 222 days [mode: 30 days (range: 2,553)], with problematic cocaine use for 7 years [mode: 1 year (range: 32 years)] and a number of DSM-IV criteria for CUDs of 8–9 (severe CUD). In addition to cocaine, there was a high prevalence of patients diagnosed with other substance use disorders (68%).

**Table 1:** Characteristics related to cocaine use in abstinent CUD patients.

Variable		Cocaine Group
Current cocaine abstinence (duration) [mean (SD)]	*days*	221.8 (429.4)
Problematic cocaine use (duration) [mean (SD)]	*years*	7.34 (6.47)
DSM-IV criteria for cocaine abuse and dependence (CUD severity) [mean (SD)]	(0–11)	8.4 (2.2)
Comorbid substance use disorders [N (%)]	No	32 (32.0)
	Yes	68 (68.0)

We evaluated the relative effects of abstinence, problematic cocaine use and DSM-IV criteria for CUDs on plasma TRP-derived metabolites in the cocaine group using ANCOVA and controlling for ‘sex’, ‘age’ and ‘BMI’. However, we found no effects or associations between these descriptive variables and the expression of these metabolites (data not shown).

### Psychiatric comorbidity and effects of mental disorders on plasma concentrations of TRP-derived metabolites in abstinent CUD-patients

The cocaine group exhibited a high prevalence of psychiatric comorbidity (about 82%), considering comorbid mental disorders and comorbid substance use disorders, such that common mental disorders were diagnosed in 59% of CUD-patients. We divided patients into comorbid and non-comorbid patients according to lifetime diagnosis of mental disorders for further characterization (Table 2). We found that abstinent CUD-patients diagnosed with comorbid mental disorders had higher psychotropic medication use (p < 0.001) and higher prevalence of other substance use disorders (p < 0.05) than in patients with no comorbid mental disorders.

**Table 2:** Psychiatric characteristics of abstinent CUD-patients grouped according to diagnosis of comorbid mental disorders.

Variable		Comorbid Mental Disorders		
		NO N = 41	YES N = 59	P-value
Age [mean (SD)]	*years*	34.5 (7.9)	36.1 (7.2)	ns^**a**^
Body mass index [mean (SD)]	*kg/m*^2^	25.6 (3.9)	25.3 (4.8)	ns^**a**^
Sex [N (%)]	Female	4 (9.8)	14 (23.7)	ns^**b**^
	Male	37 (90.2)	45 (76.3)	
Prescribed psychotropic medication use [N (%)]	No	24 (58.5)	14 (23.7)	<0.001^**b**^
	Yes	17 (41.5)	45 (76.3)	
Psychological/psychiatric treatment (no substance-related) [N (%)]	No	32 (78.0)	25 (42.4)	<0.001^**b**^
	Yes	9 (22.0)	34 (57.6)	
	Outpatient	9 (22.0)	31 (52.5)	—
	Income	0 (0.0)	3 (5.1)	
Treatment for substance use disorders [N (%)]	No	10 (24.4)	4 (6.8)	<0.05^**b**^
	Yes	31 (75.6)	55 (93.2)	
	Outpatient	29 (70.7)	47 (79.7)	—
	Income	2 (4.9)	8 (13.6)	
Lifetime substance use disorders (excluding cocaine) [N (%)]	No	18 (43.9)	14 (23.7)	<0.05^**b**^
	Yes	23 (56.1)	45 (76.3)	
	Alcohol	21 (51.2)	38 (64.4)	—
	Cannabis	4 (9.8)	13 (22.0)	
	Heroin	0 (0.0)	9 (15.3)	
	Sedatives	3 (7.3)	8 (13.6)	
	Others	2 (4.9)	7 (11.9)	
Common lifetime mental disorders [N (%)]	No	41 (100.0)	0 (0.0)	<0.001^**b**^
	Yes	0 (0.0)	59 (100.0)	
	Mood	—	28 (47.5)	—
	Anxiety	—	22 (37.3)	
	Psychosis	—	16 (27.1)	
	Personality	—	38 (64.4)	

^a^P-value from Student’s t-test.

^b^P-value from Fisher’s exact test or Chi-square test.

Abbreviations: ns, non-significant.

Because TRP and 5-HT have been linked to psychiatric disorders, we studied the effect of common ‘comorbid mental disorders’ on TRP and metabolites in the plasma of the cocaine group. These analyses were controlled for ‘sex’, ‘age’ and ‘BMI’ but also for ‘psychiatric medication’ (during the last year) as confounding variables. A base-10 logarithmic transformation of estimated marginal means was conducted for QA and ratios.

**Figure 2:**
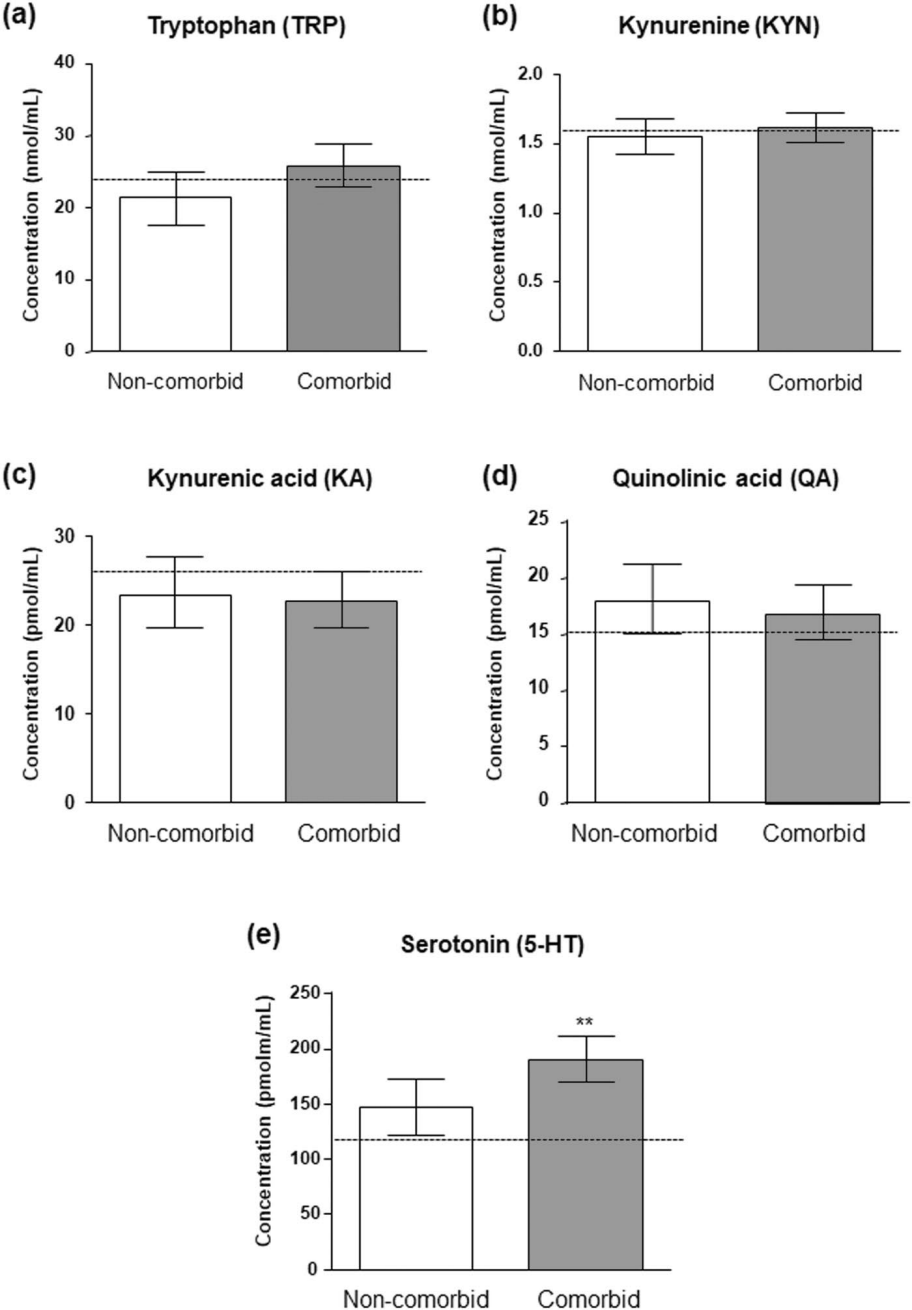
Plasma concentrations of TRP-derived metabolites in abstinent CUD-patients according to diagnosis of comorbid mental disorders. Plasma concentrations of **(a)** TRP, **(b)** KYN, **(c)** KA, **(d)** QA and **(e)** 5-HT were determined in the non-comorbid subgroup and the comorbid subgroup (common mental disorders: mood disorders, anxiety, psychotic disorders and personality disorders). Bars are estimated marginal means and 95% CI. Data were analyzed by ANCOVA. (**)p < 0.01 denotes significant differences compared with the non-comorbid subgroup. Dashed lines indicate marginal means of the control group.

As shown in Fig. 2, we observed no effect of ‘comorbid mental disorders’ on the expression of TRP metabolism in the plasma (Fig. 2a–d) with the exception of 5-HT. Thus, statistical analysis revealed a significant main effect of ‘comorbid mental disorders’ (F_(1,93)_ = 7.539, p = 0.007; Fig. 2e) on 5-HT concentrations, and patients with comorbid mental disorders had increased 5-HT concentrations relative to non-comorbid patients [188.25 (95% CI = 167.24–209.28) pmol·mL^−1^ and 146.86 (95% CI = 120.16–173.57) pmol·mL^−1^, respectively]. Furthermore, the covariate ‘age’ was related to 5-HT concentrations (F_(1,93)_ = 6.961, p = 0.010) and correlation analysis indicated a negative association between age and 5-HT concentrations (r = −0.199, p < 0.05) in the cocaine group, although the association was not significant in each subgroup (non-comorbid or comorbid patients) (Fig. S2). As previously indicated, ‘BMI’ was found to be related to KYN concentrations (F_(1,93)_ = 13.323, p < 0.001).

With regard to the ratios, we found no main effect of ‘comorbid mental disorders’ (Table S3).

### Comorbid mental disorders and plasma 5-HT concentrations in abstinent CUD-patients

As a continuation of the assessment of the effects of comorbid mental disorders on plasmatic 5-HT concentrations, we evaluated the contribution of the most prevalent mental disorders in the cocaine group to the observed effect: mood disorders (N = 28), anxiety (N = 22), psychotic disorders (N = 16) and personality disorders (i.e., borderline and antisocial personality disorders) (N = 38).

**Figure 3:**
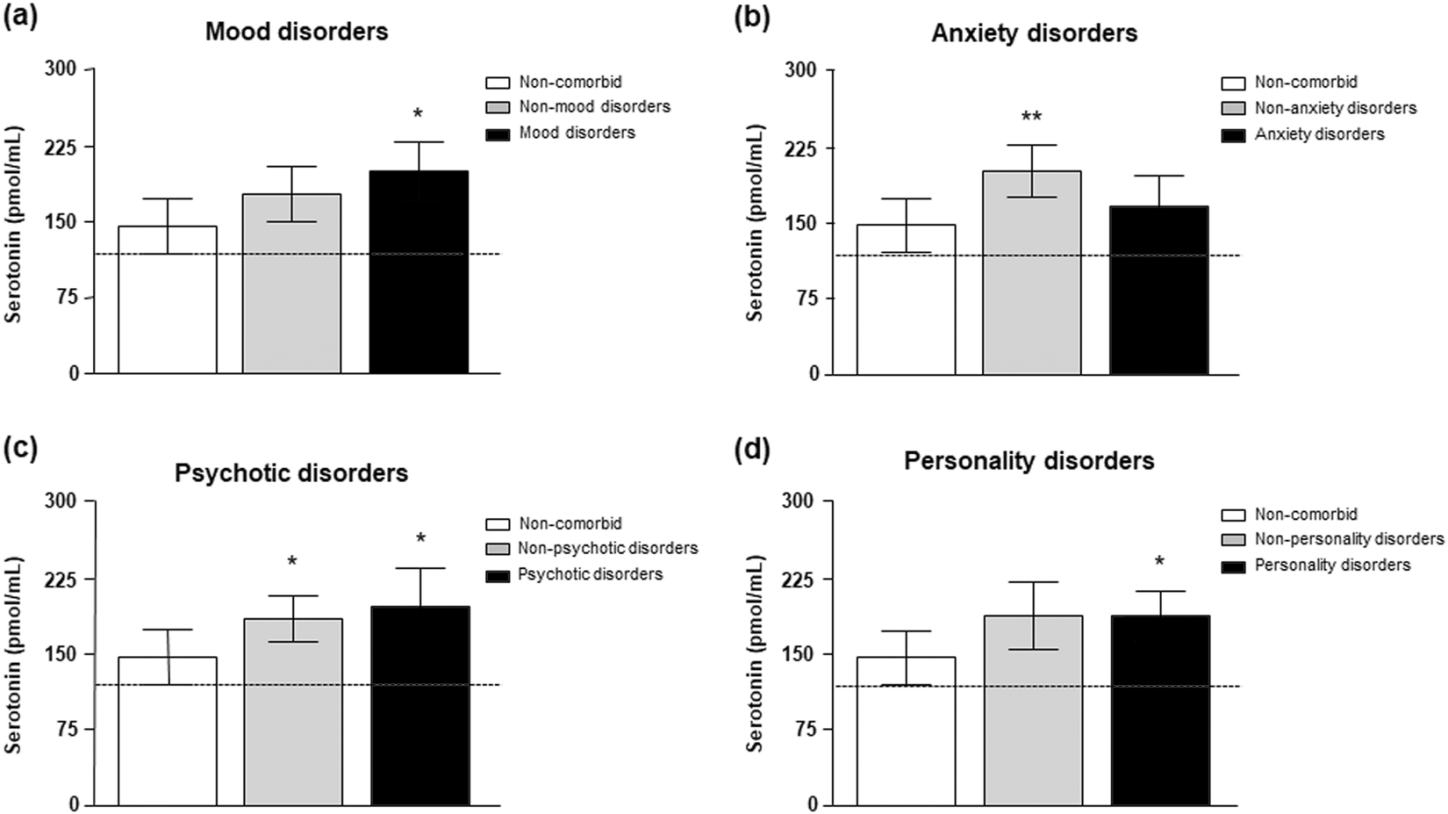
Plasma concentrations of 5-HT in abstinent CUD-patients according to diagnosis of common mental disorders. mood, anxiety, psychotic and personality disorders. Plasma concentrations of 5-HT were determined in the non-comorbid subgroup and other comorbid subgroups: (**a**) Mood disorders; (**b**) Anxiety disorders; (**c**) Psychotic disorders; and (**d**) Personality (borderline and antisocial) disorders. Bars are estimated marginal means and 95% CI. Data were analyzed by ANCOVA. (**)p < 0.01 and (**)p < 0.01 denote significant differences compared with the non-comorbid subgroup. Dashed lines indicate marginal means of the control group.

We analyzed separately the effects of mental disorders on 5-HT concentrations using ANCOVA and including as primary factor the diagnosis of each comorbid mental disorder with 3 categories/subgroups [non-comorbid subgroup or lack of any mental disorder (N = 41), non-mental disorder subgroup or lack of the particular mental disorder and comorbid mental disorder subgroup or diagnosis of the particular mental disorder]. Again, ‘sex’, ‘age’, ‘BMI’ and ‘psychiatric medication’ were controlled as confounding variables. Estimated marginal means of 5-HT concentrations for each subgroup were represented in Fig. 3.

Overall, there was a significant main effect of each mental disorder on plasma 5-HT concentrations. However, although 5-HT concentrations were significantly affected by ‘mood disorders’ (F_(2,92)_ = 4.581, p = 0.012), ‘anxiety disorders’ (F_(2,92)_ = 5.581, p = 0.005), ‘psychotic disorders’ (F_(2,92)_ = 3.930, p = 0.022) and ‘personality disorders’ (F_(2,92)_ = 3.729, p = 0.028), *post hoc* comparisons revealed differences when CUD-patients diagnosed with these comorbid mental disorders were compared with non-comorbid patients. Thus, the estimated 5-HT mean was significantly higher in CUD-patients diagnosed with mood (Fig. 3a), psychotic (Fig. 3c) and personality (Fig. 3d) disorders than in non-comorbid patients with no mental disorders (p < 0.05). In contrast, abstinent CUD-patients diagnosed with anxiety disorders showed 5-HT concentrations similar to non-comorbid patients (Fig. 3b).

In summary, we observed higher 5-HT concentrations in abstinent CUD-patients diagnosed with lifetime mental disorders, particularly with mood, psychotic and personality disorders. In fact, the highest concentrations of 5-HT were detected in CUD-patients diagnosed with only mood disorders (N = 9): 188.22 (95% CI = 118.60–257.85) pmol·mL^−1^, whereas patients with only psychosis (N = 7) [159.73 (95% CI = 89.47–229.96) pmol·mL^−1^] or personality disorders (N = 9) [153.31 (95% CI = 109.69–196.92) pmol·mL^−1^] displayed lower concentrations of 5-HT.

## Discussion

The identification of biomarkers in cocaine addiction is a clinical need that might help to stratify patients on the basis of their clinical phenotype and response to treatment. In this study we evaluated TRP metabolism in the plasma of abstinent cocaine users diagnosed with CUD to identify metabolites linked to the cocaine addict phenotype. We report a decrease in the plasma concentration of KA, a neuroprotective metabolite of the KYN pathway, which is independent of comorbid mental disorders and therefore attributable to CUD. We observed no significant differences between groups in TRP or KYN concentrations, and consequently there were no differences in KYN/TRP ratios indicating that TDO/IDO activity was unchanged in cocaine users. Finally, we also report an increase in plasma 5-HT concentration in abstinent CUD-subjects, which appears to be dependent on the presence of comorbid mood and personality disorders.

To date, studies examining differences in TRP metabolites between cocaine-dependent and control subjects are limited. A previous metabolomics study reported differences in various metabolites of the TRP, tyrosine and purine pathways in the plasma of cocaine-dependent subjects with at least two weeks of abstinence^15^. In contrast to our results, they reported no differences in 5-HT concentration although they did observe an increase in N-methyl-serotonin, a minor metabolite of 5-HT. The authors excluded patients diagnosed with mental disorders and since the changes we observe in 5-HT in the cocaine group depend on the presence of comorbid mental disorders, this could explain the difference in results. It is also very important to emphasize that we analyzed plasma 5-HT after a protracted period of abstinence.

In the periphery, 5-HT is synthesized by means of the rate-limiting enzyme tryptophan hydroxylase and secreted into blood by enterochromaffin cells of the intestine^16^. Since 5-HT is a charged molecule that cannot cross the blood-brain barrier, its concentration is regulated independently in each compartment. Given the role of SERT in regulating 5-HT concentration and the close relationship that exists between platelet SERT and plasma 5-HT it is reasonable to propose that platelet SERTs are involved in the elevation of plasma 5-HT in abstinent patients. Specifically, plasma membrane SERT, as well as 5-HT uptake, initially rise as platelets are exposed to increasing 5-HT concentration, but this initial response is followed by a second phase, in which higher concentrations of 5-HT decrease SERT levels^17^. In fact, it has been reported that a reduction in platelet SERT density in cocaine-dependent individuals could be the consequence of maintained high concentrations of 5-HT^18–20^.

The expression of SERT is controlled by a highly-conserved single gene (in humans *SLC6A4* gene) and SERT expressed in platelets and 5-HT neurons appear to be identical^21^. Thus, the results obtained in the periphery may reflect changes that occur in the brain although any extrapolation must be made with due caution.

In this sense, genetic factors should be taken into account since the short variant of SERT polymorphism termed the 5-HT transporter-linked polymorphic region (*h5*-*HTTLPR)* reduces the transcriptional efficiency of the gene, resulting in decreased SERT expression^22,23^. This polymorphism has been linked to some psychiatric disorders such as major depressive disorder^24–26^ although the findings are controversial. Whether a higher incidence of the short allele of the transporter gene was present in the CUD-patients with comorbid psychiatric disorder of our study needs to be explored.

In addition, regulation of SERT transcription in intestinal epithelium differs from that for neuronal SERT^27^, so we cannot rule out a possible role of this factor in the effect observed however, their involvement in regulating plasma 5-HT concentration is difficult to establish^28^.

Finally, because plasma 5-HT results from equilibrium among synthesis, platelet uptake and storage, and metabolism, we cannot rule out that 5-HT concentrations might be increased not only by changes in uptake, as suggested above, but also by changes in synthesis or metabolism. These possibilities should be explored in future studies. Interestingly, in abstinent CUD-patients we found a negative correlation between 5-HT concentration and age which appears to be independent of the presence or not of comorbid mental disorders, indicating that younger CUD-subjects showed greater increases in 5-HT concentration than older subjects. It is tempting to speculate that these greater increases in 5-HT in younger patients might be related to a shorter duration of cocaine abuse although other factors such as recent intensity of cocaine consumption might explain this observation. In any case, considering that both platelet 5-HT uptake and plasma 5-HT concentrations are clearly influenced by age, sex and season^29,30^, this hypothesis needs to be conclusively demonstrated.

With regard to the changes observed in the KYN pathway, no association with comorbid disorders was found for any of the metabolites analysed. Previous studies had pointed to alterations of kynurenine pathway linked to depression, as well as to schizophrenic or bipolar patients. However, the results from human studies are not conclusive and sometimes contradictory since the findings are dependent on the stage of the illness, age, brain anatomic area involved and sometimes even contradictory between peripheral and central nervous system^31,32^. Furthermore, it must be considered that cocaine addiction in the patients of our study may be contributing to the lack of changes associated with comorbidity and, as mentioned below, one of the limitations of this study is that data from patients with mental disorders without cocaine addiction are missing.

It is interesting that the decrease in serum concentration of KA in CUD-patients occurs in the absence of changes in KYN concentration pointing to the possible involvement of a downstream imbalance in the KYN metabolic pathway. KA is formed from KYN by the action of kynurenine amino-transferases (KATs) and thus the ratio KA/KYN can be considered an index of the activity of this group of enzymes. Our results show a decrease in this KA/KYN ratio in abstinent CUD-patients compared with control subjects. These findings suggest that either KAT enzyme levels or activity is modified in abstinent cocaine-addicted subjects and could be a consequence of endogenous KAT inhibitors^33^ or modifications of its properties by genetic mutations.

The reduced formation of KA may contribute to an imbalance in the neuroprotective and neurodegenerative pathways^10^. Previous studies have demonstrated that the concentration of 3HK (formed from KYN through the action of kynurenine 3-monooxygenase) is unaltered in CUD-patients^15^ and because we found no change in the concentration of QA, the decrease in the neuroprotective pathway might permit greater activity from this neurotoxic metabolite. However, it is important to highlight that we have no evidence of how the plasma concentrations reflect CNS concentrations although a significant correlation between peripheral and CSF levels of QA and KA have been suggested^34^.

This study indicates that cocaine addiction and its medical consequences might be associated with dysregulation in the KYN pathway. A specific enhancement of KAT activity or KA concentrations in order to return to homeostatic levels could be beneficial in cocaine abstinence and, in particular, in lowering drug craving and seeking. In this sense, recent evidence indicates that increasing KA may be an effective strategy for inhibiting the rewarding effects of THC and alcohol- and cocaine-seeking behavior and relapse in animal models^11,13^.

### Limitations

These findings support the importance of monitoring TRP metabolites in the context of CUD and psychiatric comorbidity, but we are aware of the limitations of this cross-sectional study: (i) The sources for recruitment were different in the control group and the cocaine group, therefore several socio-demographic variables can influence the association between biochemical data and diagnosis of CUD (e.g., ‘education’ and ‘occupation’); (ii) The recruitment of CUD-patients was conducted from outpatient programs and there are clinical variables that remain unknown and can affect the validity of results; (iii) Larger samples of abstinent CUD-patients and additional study groups should be included (e.g., subjects with mental disorders and no substance use); (iv) Longitudinal studies are needed to monitor changes in these metabolites during abstinence at different times in the same patients; and (v) Pharmacological and behavioral studies in animal models can help to elucidate the association between TRP metabolites and cocaine exposure.

In summary, our findings indicate the existence of changes in TRP metabolism in subjects with lifetime CUD. More specifically, KYN pathway dysregulation is a consequence of cocaine consumption while plasma 5-HT concentrations are related to comorbid psychiatric disorders in cocaine addiction. These results emphasize the need to find biomarkers that allow a better stratification of cocaine-addicted patients in order to develop therapeutic approaches to prevent cocaine relapse following periods of abstinence.

## Materials and Methods

### Ethics approval and consent to participate

Written informed consent was obtained from each participant after they had received a complete description of the present study and had been given the chance to discuss any questions or issues. The study and protocols for recruitment, privacy and confidentiality were approved by the Ethics Committee for Research of our institution (*Comité de Ética de la Investigación Provincial de Málaga*) (CP14/00212) in accordance with the Ethical Principles for Medical Research Involving Human Subjects adopted in the Declaration of Helsinki by the World Medical Association (64th WMA General Assembly, Fortaleza, Brazil, October 2013), Recommendation No. R (97) 5 of the Committee of Ministers to Member States on the Protection of Medical Data (1997), and Spanish data protection act (*Ley Orgánica* 15/1999 de *Protección de Datos*, LOPD).

### Participants and recruitment

The present cross-sectional study was conducted in accordance with the STROBE (strengthening the reporting of observational studies in epidemiology) statement^35^. The procedure for recruiting participants study was a non-random convenience sampling (June 2014 to June 2016) according to the participation criteria.

One hundred white Caucasian participants were selected in the cocaine group from outpatient treatment programs for cocaine addiction at centers for drug dependence treatment in the province of Malaga (*Centro Provincial de Drogodependencias* and *Centros Ambulatorios de Tratamiento*) (Malaga, Spain) after confirming the eligibility criteria. Sixty healthy individuals from a multidisciplinary staff working at the *Hospital Regional Universitario de Málaga* (Malaga, Spain) were included in the control group.

#### Eligibility criteria

The participation was voluntary but all participants had to meet eligibility criteria. To be eligible, subjects had to be between 18 and 65 years of age. Exclusion criteria were as follows: (1) Personal history of chronic diseases and/or cancer; (2) Infectious diseases; (3) Incapacitating cognitive alterations; and (4) Pregnancy for women.

Cocaine group: Cocaine users were required to have been diagnosed with lifetime CUD (i.e., cocaine abuse or dependence) exclusively by intranasal use and also to be abstinent from abused drugs (except for nicotine and caffeine) for at least 2 weeks before testing. Lifetime CUD was determined through psychiatric interviews, while the abstinence from abused drugs (cocaine, amphetamine, opiates, barbiturates, phencyclidine and cannabis) was checked weekly by urine analysis using a V-Twin Drug Testing System (Siemens AG, Erlangen, Germany) in the outpatient settings. Subsequently, rapid plasma analyses were conducted to verify abstinence from cocaine and alcohol.

All cocaine-addicted subjects were under treatment interventions, including pharmacological and behavioral approaches. Regarding the pharmacological interventions, 45 participants were treated with psychotropic medication(s) in the last year: anxiolytics (N = 22), antipsychotics (N = 5), antidepressants (N = 21) and disulfiram (N = 4).

Control group: The control group had no history of mental disorders or substance use disorders (lifetime abuse or dependence) and was sex-balanced and matched for age and body mass index with the cocaine group. No psychotropic medication had been used during the last year.

#### Exclusions

Thus, 110 cocaine users and 65 controls were initially recruited for this observational study based on the eligibility criteria. However, 15 participants (10 cocaine users and 5 controls) were excluded after examining for eligibility in the interviews or rapid plasma analyses.

### Clinical evaluations

Prior to the clinical assessments, the ‘Trail Making Test’ (TMT) Part B was administered to all participants as a memory and attention-screening test to detect cognitive alterations^36^.

All abstinent CUD-subjects were evaluated according to the ‘Diagnostic and Statistical Manual of Mental Disorders-4th Edition-Text Revision’ (DSM-IV-TR) criteria, using the Spanish version of the ‘Psychiatric Research Interview for Substance and Mental Diseases’ (PRISM)^37,38^. Lifetime prevalence was used to present the frequency of substance use disorders, non-substance use disorders or common mental disorders. DSM-IV-TR criteria for substance dependence and abuse were used to diagnose substance use disorders and determine the severity of CUD^14,39^.

Control subjects were initially evaluated by PRISM (for substance screening and abuse and dependence) and the Spanish version of ‘Dual Diagnosis Screening Instrument’ (DDSI) to detect psychiatric disorders^40^. All the interviews were performed by experienced psychologists who had received training for TMT, PRISM and DDSI.

### Laboratory methods

#### Collection and rapid analyses of plasma samples

Blood samples were collected (09:00–11:00 h AM) after fasting for 8–12 h (prior to the psychiatric interviews). Venous blood was extracted into 10 mL K2 EDTA tubes (BD, Franklin Lakes, NJ, USA) and was immediately processed to obtain plasma. Blood samples were centrifuged at 2,200 × g for 15 min (4 °C) and individually assayed to detect infectious diseases by 3 rapid tests for HIV, hepatitis B and hepatitis C purchased from ALL.DIAG (Strasbourg Cedex, France). Samples displaying infection were discarded following safety protocols. Plasma analyses for cocaine metabolite (Benzoylecgonine Specific Direct ELISA Kit purchased from Immunalysis, Pomona, CA, USA) were also performed to confirm cocaine abstinence. Additionally, the blood alcohol concentration was measured according to the alcohol oxidase reaction using an Analox AM1 analyzer (Analox Instruments, Stourbridge, UK).

#### Determination of TRP, 5-HT, KYN, KA and QA

Plasma aliquots (100 μL) were deproteinized with perchloric acid, and tyrosine added as internal standard. After centrifugation, supernatants were injected onto a reverse phase column (HR-80; 80 mm × 4.6 mm, 3 µm; Thermo Fisher Scientific). KYN was eluted using a mobile phase containing 0.1 M sodium acetate and 4% acetonitrile (adjusted to pH 4.6) and determined by UV detection (360 nm, Waters 2487) whereas TRP and KA were separated using a mobile phase containing 0.5 M sodium acetate (adjusted to pH 6.2), 0.25 M zinc acetate and 5% acetonitrile, and detected fluorometrically at excitation/emission wavelengths of 270/360 nm for TRP and 344/398 nm for KA (Waters 2475). 5-HT was separated using an ODS2-C18 column (150 mm × 4.6 mm, 5 µm, Waters) and a mobile phase consisting of 0.1 M sodium acetate (adjusted to pH 3.8) and 8% methanol, and detected fluorometrically at excitation/emission wavelengths of 290/337 nm.

QA was determined using a commercially available ELISA immunoassay (Cloud-Clone Corp., Houston, USA) according to instructions of the manufacturer.

The ratios of KYN or 5-HT to TRP and KA or QA to KYN were used as a measure of TRP degradation and of KA and QA formation, respectively.

### Statistical analysis

All data for tables are expressed as number and percentage of subjects [N (%)] or mean and standard deviation [mean (SD)]. The significance of differences in categorical and normal continuous variables was determined using Fisher’s exact test (Chi-square test) and Student’s t-test, respectively.

Statistical analysis of concentrations and ratios was performed using analysis of covariance (ANCOVA) as a general linear model. The ANCOVA models included CUD-related variables as categorical and continuous independent variables to explore their association with TRP, KYN, KA, QA and 5-HT concentrations. Because the size of the sample restricts the number of independent variables in the ANCOVA, only biological variables (‘sex’, ‘age’, ‘BMI’) and ‘psychiatric medication’ were used as covariates. Socio-demographic characteristics were not used as covariates in the study because they are multilevel categorical variables whose interpretation is complex, they do not meet parametric assumptions and, therefore, decrease the statistical efficiency. To ensure statistical assumptions of a general linear model, log(10)-transformation was used for positive skewed distributions. Estimated marginal means [95% confidence intervals (95% CI)] were expressed and represented in the figures, using back-transformation if there was logarithmic transformation. The *post hoc* comparisons were performed using Sidak’s correction test.

Additionally, correlation analyses were performed using the Pearson’s coefficient (r).

A p-value < 0.05 was considered statistically significant. Statistical analyses were performed using the Graph-Pad Prism version 5.04 software (GraphPad Software, San Diego, CA, USA) and IBM SPSS Statistical version 22 software (IBM, Armonk, NY, USA).

### Ethical standards

The authors assert that all procedures contributing to this work comply with the ethical standards of the relevant national and institutional committees on human experimentation and with the Helsinki Declaration of 1975, as revised in 2008.

## Supplementary information

Supplementary material
