# Supplementary material for: Serotonin is the main tryptophan metabolite associated with psychiatric comorbidity in abstinent cocaine-addicted patients
Source: Sci Rep. 2019 Nov 14;9:16842. doi: 10.1038/s41598-019-53312-0 (PMC6856167; doi:10.1038/s41598-019-53312-0)
Supplement: Supplementary file 1 — Supplementary material [file 41598_2019_53312_MOESM1_ESM.pdf]

Serotonin is the main tryptophan metabolite associated with psychiatric comorbidity in abstinent cocaine-addicted patients

Pedro Araos[1, 2$], Rebeca Vidal[3$], Esther O'Shea[3], María Pedraz[1], Nuria García-Marchena[1], Antonia Serrano[1], Juan Suárez[1], Estela Castilla-Ortega[1, 2], Juan Jesús Ruiz[4], Rafael Campos-Cloute[4], Luis J. Santín[2], Fernando Rodríguez de Fonseca[1], Francisco Javier Pavón[*1], María Isabel Colado[*3]

SUPPLEMENTARY FIGURES

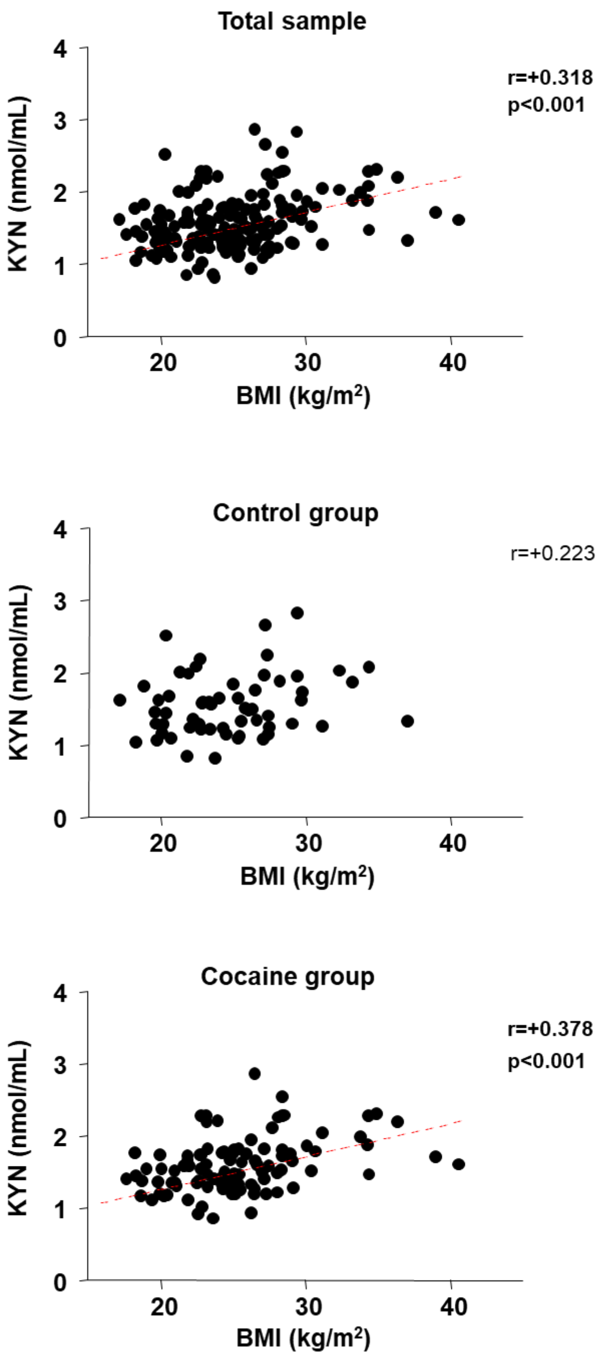

Figure S1. Correlation analyses between plasma concentrations of KYN and BMI in abstinent CUD-patients and control subjects. Black dots are individual values. Pearson correlation coefficients (r) were calculated for the total sample, the control group and the cocaine group.

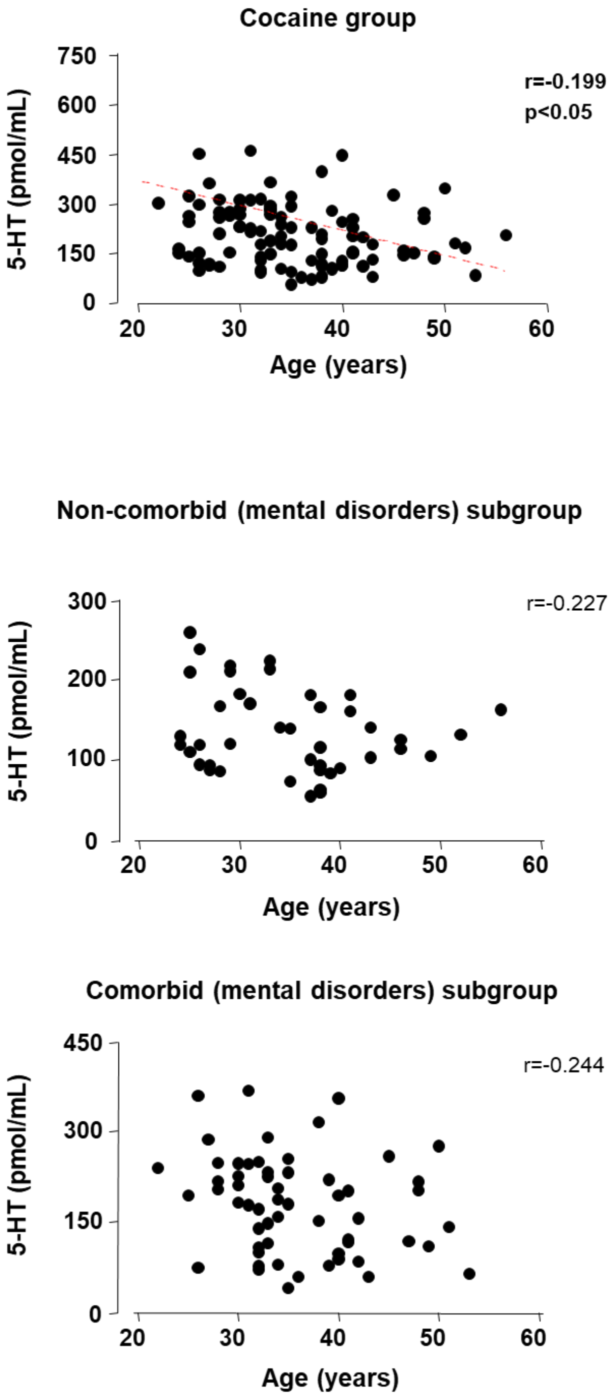

Figure S2. Correlation analyses between plasma concentrations of 5-HT and age in abstinent CUD-patients according to diagnosis of comorbid mental disorders. Black dots are individual values. Pearson correlation coefficients (r) were calculated for the control group, the non-comorbid subgroup and the comorbid (mental disorders) subgroup.

Table S1. Biological and socio-demographic variables

VARIABLE		COCAINE GROUP N=100	CONTROL GROUP N=60	P-value
**Age [mean (SD)]**	*years*	35.4 (7.5)	36.4 (10.4)	ns [a]
**Body mass index [mean (SD)]**	*kg/m^2*	25.4 (4.4)	24.6 (4.3)	ns [a]
**Sex [N (%)]**	Female<br>Male	18 (18.0)<br>82 (82.0)	30 (50.0)<br>30 (50.0)	<0.001 [b]
**Marital status [N (%)]**	Single<br>Married/cohabiting<br>Divorced/separated<br>Widowed	40 (40.0)<br>37 (37.0)<br>23 (23.0)<br>0 (0.0)	29 (48.3)<br>25 (41.7)<br>6 (10.0)<br>0 (0.0)	ns [b]
**Education [N (%)]**	≤ Primary<br>Secondary<br>Higher	57 (57.0)<br>32 (32.0)<br>11 (11.0)	5 (8.3)<br>25 (41.7)<br>30 (50.0)	<0.001 [b]
**Occupation [N (%)]**	Student<br>Employed<br>Unemployed<br>Retired	0 (0.0)<br>41 (41.0)<br>58 (58.0)<br>1 (1.0)	4 (6.7)<br>52 (86.7)<br>4 (6.7)<br>0 (0.0)	<0.001 [b]

([a]) P-value from Student's t-test.
([b]) P-value from Fisher's exact test or Chi-square test.
Abbreviations: ns, non-significant

Table S2. Ratios of metabolites related to TRP of participants.

RATIO		COCAINE GROUP	CONTROL GROUP	P-value [a]
**KYN/TRP**	**mean (95%CI)**	7.08×10^{-2} (5.92×10^{-2}-8.47×10^{-2})	7.60×10^{-2} (6.22×10^{-2}-9.31×10^{-2})	ns
**KA/KYN**	**mean (95%CI)**	1.49×10^{-2} (1.36×10^{-2}-1.63×10^{-2})	1.73×10^{-2} (1.57×10^{-2}-1.91×10^{-2})	**0.022**
**QA/KYN**	**mean (95%CI)**	11.02 (9.73-12.47)	9.42 (8.47-10.50)	ns
**5-HT/TRP**	**mean (95%CI)**	2.39×10^{-3} (2.07×10^{-3}-2.81×10^{-3})	1.94×10^{-3} (1.70×10^{-3}-2.25×10^{-3})	**0.046**

([a]) P-values from ANCOVA (*F* statistics and degrees of freedom are indicated in the text).
Abbreviations: ns, non-significant

Table S3. Ratios of metabolites related to TRP metabolism of abstinent CUD-patients according to comorbid mental disorders.

RATIO		COMORBID MENTAL DISORDERS		P-value [a]
		NON-COMORBID	COMORBID	
**KYN/TRP**	**mean (95%CI)**	8.89×10^{-2} (6.68×10^{-2}-11.83×10^{-2})	6.56×10^{-2} (5.24×10^{-2}-8.22×10^{-2})	0.063
**KA/KYN**	**mean (95%CI)**	1.52×10^{-2} (1.31×10^{-2}-1.76×10^{-2})	1.43×10^{-2} (1.27×10^{-2}-1.61×10^{-2})	0.772
**QA/KYN**	**mean (95%CI)**	10.67 (8.63-13.15)	10.45 (8.77-12.46)	0.855
**5-HT/TRP**	**mean (95%CI)**	3.36×10^{-3} (2.47×10^{-3}-4.56×10^{-3})	3.06×10^{-3} (2.40×10^{-3}-3.88×10^{-3})	0.582

([a]) P-values from ANCOVA (*F* statistics and degrees of freedom are indicated in the text).
